# Gut Microbiota-Immune System Crosstalk and Pancreatic Disorders

**DOI:** 10.1155/2018/7946431

**Published:** 2018-02-01

**Authors:** D. Pagliari, A. Saviano, E. E. Newton, M. L. Serricchio, A. A. Dal Lago, A. Gasbarrini, R. Cianci

**Affiliations:** ^1^Department of Gastroenterology, Pancreatic Unit, Catholic University of the Sacred Heart School of Medicine, Rome, Italy; ^2^Department of Internal Medicine, Catholic University of the Sacred Heart School of Medicine, Rome, Italy; ^3^CytoCure LLC, Beverly, MA, USA

## Abstract

Gut microbiota is key to the development and modulation of the mucosal immune system. It plays a central role in several physiological functions, in the modulation of inflammatory signaling and in the protection against infections. In healthy states, there is a perfect balance between commensal and pathogens, and microbiota and the immune system interact to maintain gut homeostasis. The alteration of such balance, called dysbiosis, determines an intestinal bacterial overgrowth which leads to the disruption of the intestinal barrier with systemic translocation of pathogens. The pancreas does not possess its own microbiota, and it is believed that inflammatory and neoplastic processes affecting the gland may be linked to intestinal dysbiosis. Increasing research evidence testifies a correlation between intestinal dysbiosis and various pancreatic disorders, but it remains unclear whether dysbiosis is the cause or an effect. The analysis of specific alterations in the microbiome profile may permit to develop novel tools for the early detection of several pancreatic disorders, utilizing samples, such as blood, saliva, and stools. Future studies will have to elucidate the mechanisms by which gut microbiota is modulated and how it tunes the immune system, in order to be able to develop innovative treatment strategies for pancreatic disorders.

## 1. Introduction

The human gastrointestinal tract hosts more than 10^14^ microorganisms, a number 10 to 20 times greater than the total number of cells of the human body, and includes at least 1000 different microbial species, including bacteria, fungi, yeast, viruses, and archaea [1–3]. The ensemble of these populations constitutes the so-called gut microbiota. Instead, the collection of the whole genome sequence of gut microbiota species is called microbiome and consists of more than 5,000,000 genes [4–7].

Gut microbiota is central to the development and modulation of the mucosal innate and adaptive immune system and exerts an important role in the protection against pathogenic microbes by maintaining gut integrity and regulating intestinal barrier permeability. It weighs about 900–1200 g and participates in several physiological functions. Indeed, gut microbiota is constantly involved in facilitating digestion, storing nutrients, secreting vitamins, activating metabolic functions, and shaping intestinal architecture [8]. It is composed of various microbial populations, the most prevalent being the Firmicutes and Bacteroidetes phyla which together represent about 80–90% of the whole gut microbiota [9]. These microbial populations are separated from intestinal epithelial cells by a physical-chemical barrier composed of mucus, mucin glycoproteins, and multiple antibacterial molecules, including alpha-defensins, C-type lectins, lysozyme, phospholipase A2, and secretory IgA [10]. In healthy conditions, all gut microbial species are in a mutualistic or commensal symbiotic state contributing to a perfect and constant homeostasis [11]. In such state, the interaction between gut microbiota, intestinal epithelial cells, and the mucosal immune system creates an environment which controls overgrowth of the host pathogenic flora [12] and limits the colonization of the intestinal tract by foreign pathogens [13–16].

The breakdown of this balance between gut microbiota, the immune system, and the intestinal epithelial barrier results in a pathological condition called dysbiosis [17]. In recent years, several diseases and dysfunctions have been linked to intestinal dysbiosis, including celiac disease, inflammatory bowel disease (IBD), and irritable bowel syndrome (IBS), as well as other conditions [18–24]. In a similar way, given that pancreas is known not to have its own microbial collection, gut microbiota may be involved in the pathogenesis of pancreatic disorders [25]. In this article, we will review the currently available data linking gut microbiota-immune system crosstalk and several pancreatic disorders, such as pancreatitis, diabetes, and pancreatic cancer.

## 2. Inflammatory Pancreatic Diseases

Acute pancreatitis is an inflammatory disease frequently associated with gallstones or alcohol consumption with a high risk of mortality.

Chronic pancreatitis, instead, is a long-standing, inflammatory disease leading to severe alterations in pancreatic structure and function. The typical clinical manifestations are recurrent episodes of acute pancreatitis in a previously compromised pancreatic gland or a pancreatic exocrine insufficiency due to persistent chronic damage [26].

In either acute or chronic pancreatitis, several alterations in gut microbiota composition have been reported [27].

### 2.1. Acute Pancreatitis

Hallmark of an acute pancreatitis is an inflammatory state [28, 29] due to an imbalance between pro- and anti-inflammatory cytokines. Recently, Chen et al., in a necrotizing pancreatitis mouse model, demonstrated an overexpression of several proinflammatory cytokines and chemokines, such as TNF-alpha, IL-1beta, IL-6, IL-17A, CXCL1, and IL-18, and a parallel decrease in the Paneth cell-related antimicrobial peptides, such as alpha-defensins and lysozyme [30, 31].

Indeed, pancreatic acinar and Paneth cell-related antimicrobial peptides are essential for gut homeostasis, intestinal immunity integrity, and even for the control of microbiome composition [32]. Recently, in a mouse model, Ahuja et al. have demonstrated that deletion of the Ca^2+^ channel Orai1 in pancreatic acinar cells (Orai1^−/−^ mice) induces several signs of gut inflammation and bacterial overgrowth, leading to bacterial translocation, systemic infection, and death [33]. These experimental findings further confirm the critical role played by antimicrobial pancreatic secretion in modulating gut/pancreatic homeostasis and gut immune system integrity.

As response to inflammation-mediated tissue damage, acinar pancreatic cells produce several molecules that may have the function of damage-associated molecular patterns (DAMPs) [34], such as high-mobility group box protein 1 (HMGB1), heat shock protein 70 (Hsp70), cytosolic protease-caspase 1, nucleotide-binding domain (NLRP3), adenosine triphosphate (ATP), and DNA [35–37]. DAMPs promote activation of the Toll-like-receptors (TLRs) germline-encoded type I transmembrane receptors present on epithelial cells, immune cells, macrophages, and other cells. TLRs act as pathogen recognition receptors (PRRs) and are able to identify pathogen-associated molecular patterns (PAMPs) [38]. To date, in humans, a total of at least 10 different TLRs have been recognized [39]. The TLRs most frequently implicated in the interactions with intestinal bacteria are TLR2 and TLR4, but several other TLRs may be implicated in the pathogenesis of acute pancreatitis [38, 40]. Nishio et al. demonstrated that in mice genetically deficient in the anti-inflammatory cytokine IL-10, the repeated administration of TLR4 and TLR9 ligands was able to induce pancreatic injury [41]. Matas-Cobos et al. comparing 269 acute pancreatitis patients to 269 healthy controls demonstrated that polymorphisms in TLR3 and TLR6 genes were associated with increased severity of pancreatitis [42].

Each TLR responds to distinct DAMPs, leading to the activation of specific intracellular signaling pathways, and to the production of inflammatory cytokines and chemokines [43]. Notably, in the blood of severe acute pancreatitis patients, an increase of TNF-alpha, IL-1, IL-6, and IL-10 has been documented [28, 29]. However, TLR activation is also linked to the transcription of several genes related to some nuclear factors, such as nuclear factor kappa-B (NF-kB), MAP kinase p38, JNK, and IRF-3, crucial in the control of infection and inflammation [11]. Thus, TLRs may be initially responsible for the inflammatory state, but subsequently, they protect the host, repair damaged tissue, and promote a mucosal immune response [38].

Recently, Watanabe et al. proposed that pancreatitis should be thought as a unique form of immune-mediated inflammation [44]. In this model, a pivotal role is played by TLRs (activated by pathogens related DAMPs), in inducing NF-kB-related adaptive immune system cytokines. In this proinflammatory context, damaged acinar cells begin to produce the proinflammatory cytokine IL-33 that, in turn, determines the activation and recruitment of T-cell subpopulations which participate in pancreatic inflammation.

**Figure 1:**
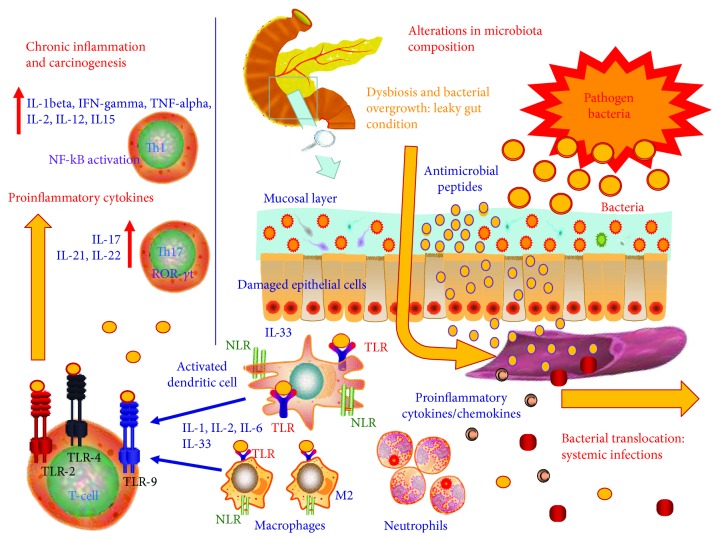
Role of leaky gut in pancreatic inflammation and carcinogenesis. The breakdown of the relationship among physiologic and pathogenic bacteria, the immune system, and intestinal epithelial barrier leads to dysbiosis. The pancreas does not possess its own microbiota, and thus, inflammatory and neoplastic processes affecting the gland may be linked to intestinal dysbiosis. In this way, during bacterial overgrowth, leaky gut is responsible for the translocation of bacteria and toxins to the pancreas. Bacterial translocation is involved in pancreatic inflammation due to toxin diffusion and complications like fibrosis, digestive and absorption disorders, diabetes, or cancer. TLR: Toll-like receptors; NLRs: NOD-like receptors; IL: interleukin; IFN: interferon; TNF: tumor necrosis factor; ROR-*γ*t: RAR-related orphan receptor-gamma t; NF-kB: nuclear factor kappa-B.

In the context of acute pancreatitis, the inflammation produces intestinal damage by several concomitant pathogenic mechanisms, such as alterations in microcirculation, vasoconstriction in the splanchnic district, and ischemia-reperfusion damage [45, 46]. This, in turn, alters intestinal permeability and leads to a condition known as leaky gut (Figure 1). When there is bacterial overgrowth, leaky gut facilitates the translocation of bacteria and toxins to the pancreas. This worsens pancreatic inflammation resulting in further damage leading to fibrosis or even, in severe cases, necrosis. The bacterial translocation may also be responsible for secondary infections that are associated with a high mortality risk [47].

Moreover, several studies have investigated the relation between inflammatory patterns and microbiota composition during acute pancreatitis. In general, during acute pancreatitis, there is an increase of pathogenic bacteria of the Enterobacteriaceae and Firmicutes families and a decrease of beneficial Bacteroidetes and Lactobacillales [28]. Gerritsen et al. in a mouse model documented that the normal intestinal flora is replaced by an “acute pancreatitis-associated microbiota” [30]. In 2015, Tan et al. published the results of a multicentre prospective clinical study involving 108 acute pancreatitis patients compared to healthy controls [28]. The authors analyzed the 10 predominant bacteria and measured several serum markers of systemic inflammation, such as plasma endotoxin, TNF-alpha, IL-1, IL-6, and IL-10. The findings have shown that the pathogenetic *Enterococcus*, of the phylum Firmicutes (order Lactobacillales), is increased while *Bifidobacterium*, of the phylum Actinobacteria (order Bifidobacteriales), is decreased. Additionally, IL-6 serum levels correlated directly with Enterobacteriaceae and *Enterococcus* number and inversely with the *Bifidobacterium* and *Clostridium* cluster XI number. The study by Tan et al. was also able to demonstrate that the extent of gut microbiota modifications predicts pancreatitis severity and the occurrence of systemic complications.

It is notable that in the context of acute pancreatitis several commensal bacteria populations have also been identified. These are associated with reduced levels of inflammatory cytokines, such as IL-1beta, TNF-alpha, CXCL1, and IL-18, and are inversely correlated with pancreatitis severity and systemic infectious complications. Thus, it can be hypothesized that the restoration of a physiological gut microbiota composition may be a useful strategy to treat acute pancreatitis [48]. Indeed, the use of probiotics in this clinical setting has been tested, but results are controversial [49]. Qin et al. in 76 acute pancreatitis patients demonstrated that the restoration of a physiological commensal/pathogens *ratio* is able to limit the systemic infectious complications [50]. On the other hand, in several other studies, oral administration of probiotics showed no significant impact on disease outcome or on the prevention of complications [48, 51, 52].

### 2.2. Chronic Pancreatitis

Chronic pancreatitis results from a long-standing inflammation leading to a chronic damage and severe functional impairment of the gland [53, 54].

It has been reported that about one-third of chronic pancreatitis patients are affected by intestinal bacterial overgrowth but the specific alterations in microbiota composition are not yet fully known [55–59]. Some authors have observed an increase in Firmicutes and a relative decrease in Bacteroidetes [27]. Recently, Jandhyala et al. published a study analyzing three groups of patients: chronic pancreatitis with and without diabetes and healthy controls. Regardless of diabetes, in pancreatitis patients, it was documented a progressive, duration-dependent reduction of the commensal bacteria *Faecalibacterium prausnitzii* [27]. Notably, *Faecalibacterium prausnitzii* promotes the homeostasis of intestinal epithelium favoring mucin production and tight-junction protein synthesis [60], induces the anti-inflammatory cytokine IL-10 [61], and regulates gut T-cell responses. Thus, the progressive reduction in *Faecalibacterium prausnitzii* observed in chronic pancreatitis patients testifies to a duration-dependent disruption of gut mucosal integrity [27]. Furthermore, *Faecalibacterium prausnitzii* levels negatively correlated with plasma endotoxin ones and an increase of endotoxin levels was associated with an impairment of glucose metabolism. Thus, the reduction in *Faecalibacterium prausnitzii* observed in chronic pancreatitis patients is an additional factor favoring the onset of diabetes or worsening its course. Then, Jandhyala et al. reported a reduction of *Ruminococcus bromii* in chronic pancreatitis patients [27]. *Ruminococcus bromii* has an important physiologic role in the degradation of starch in human colon [62]. Its reduction is related to the disruption of the gut mucosal barrier and is responsible of an alteration of the glucose metabolism.

In other studies, a reduction of *Bacteroidetes*, a Gram-negative bacteria source of lipopolysaccharide (LPS), has consistently been reported. LPS is considered a potent mediator of inflammation. In fact, in binding TLR4, LPS may activate NF-kB-related proinflammatory cytokine production [63]. Chronic pancreatitis patients have higher LPS and endotoxin levels than healthy controls, and these correlate with disease duration. LPS may induce an impairment of pancreatic beta-cells further worsening glucose metabolism [64]. The inflammatory process targets pancreatic islets, and also, T-cell recruitment occurs. In this way, literature data testifies that during chronic pancreatitis there is an increase in both Th1 and Th17 cells [65] and their related proinflammatory cytokines, such as IFN-gamma in pancreatic islets [66].

### 2.3. Autoimmune Pancreatitis

Pancreatic inflammation may elicit an immune response in the exocrine tissue, leading to either acute or chronic damage. Autoimmune pancreatitis (AIP) accounts for about 5% of all pancreatitis, and it is usually associated with other autoimmune diseases [67]. An increase in serum immunoglobulin G4 (IgG4) is a diagnostic criterion [68]. While genetic factors have been hypothesized [69], the pathogenesis of AIP remains unknown [70].

Interestingly, the gastric *Helicobacter pylori* infection has been shown to be associated with AIP [71, 72]. This bacterium is known to trigger immune responses against host tissues via several molecular mimicry pathways [73]. Guarneri et al. reported a homology between the human carbonic anhydrase II (CA-II) and alpha-carbonic anhydrase of *Helicobacter pylori* (HpCA). CA-II is an enzyme of the pancreatic epithelium whose specific serum antibodies are characteristics of AIP, and the bacterial homolog segments contain the binding motif of the high-risk HLA-DR alleles. These data demonstrated that *Helicobacter pylori* may trigger AIP in genetically predisposed subjects [74].

Other suggestions link bacterial infections with the development of AIP. In a mouse model, *Escherichia coli* induces a severe pancreatic inflammation and fibrosis similar to the human AIP [75]. Numerous studies have reported that specific microbial antigens may trigger the development of AIP activating immune responses. Gram-negative bacteria-associated LPS is able to activate immune response via-TLRs [41]. Several TLRs (TLR2, TLR3, TLR4, TLR5, and TLR7) have been linked with the development of AIP [76–78]. Among these, TLR3 typically recognizes microbial dsRNA activating the Fas/FasL-mediated cytotoxicity, responsible for chronic inflammation [79]. Finally, TLR7 is able to recognize viral ssRNA, thus activating proinflammatory signaling cascades [80].

## 3. Diabetes

### 3.1. Type 1 Diabetes

Type 1 diabetes (T1D) is characterized by a loss of insulin secretion due to damage to pancreatic beta-cells caused by an autoimmune process triggered by microbial infections.

Several alterations in gut microbiota composition have been related to the development of T1D. In a recent study on 76 children at high genetic risk, it has been demonstrated that early changes in gut microbiome composition predict T1D onset [81]. In particular, in the microbiome of these T1D predisposed children, *Bacteroides dorei* and *Bacteroides vulgatus* are increased. Instead, in people with late-onset T1D, there is not only a similar increase in *Bacteroides* species but also a reduction of *Clostridium leptum* [38, 82].

Furthermore, several bacterial or viral antigens recognized in children and teenagers have been associated later to the development of T1D [83], including antigens from *Coxsackievirus* A and B, *Echovirus*, *Enterovirus*, and so forth.

During the course of T1D, profound alterations in gut microbiota composition and related metabolites take place [84, 85]. Of importance, changes in the *ratio* of butyrate-producing *Bacteroidetes* and *Firmicutes* bacteria occur [86–88]. Other butyrate-producing and mucin-degrading bacteria, such as *Prevotella* and *Akkermansia muciniphila*, are decreased [89] while short-chain fatty acid- (SCFAs-) producing bacteria such as *Klebsiella* are increased.

Recently, Semenkovich et al. demonstrated bidirectional relationships between gut microbiota alterations and T1D-related inflammation. In fact, in a NOD mouse model, gut microbiota was able to instruct hormonal changes in the testosterone axis (in males) which led to T1D susceptibility, and the hormonal levels, in turn, were able to alter the microbial niches in the gut. This phenomenon may be a possible explanation for the different susceptibility between sexes [84, 90].

In a murine T1D model associated with a reduction in *Lactobacillus* and *Bifidobacterium* species [91], a coexisting high-grade lymphopenia [92] and an upregulation of Th17 cells have been shown [93]. These findings lend support to the hypothesis that alterations in gut microbiota composition are associated with abnormalities of the mucosal immune system and that both mechanisms participate in T1D pathogenesis [94]. In addition, a leaky gut exacerbates T1D either indirectly via beta-cell damage, due to bacterial translocation and related antigen presentation [95], or directly via beta-cell function impairment mediated by microbial toxins, such as streptozotocin [94].

Diet modification and pharmacological treatment have been similarly studied. Recently, a nonobese diabetic mouse study found that exposure to acidified water is able to increase the presence of mucosal and spleen T-regulatory cells (Tregs) and to decrease Th17 cells, thus decreasing the onset of T1D [96]. A mouse model revealed that insulin treatment is able to somewhat restore microbial populations, positively modulating the microbiota composition towards the normal, healthy state [97]. Xenobiotics have also been implicated in the pathogenesis of T1D. In a recently published study, the neonatal oral administration of vancomycin in a nonobese diabetic mouse reduced the presence of several major *genera* of Gram-positive and Gram-negative bacteria, with one single species *(Akkermansia muciniphila)* becoming dominant [98].

Furthermore, in T1D pathogenesis, a special role is played by mucosal innate and adaptive immunity. To elucidate the role of innate immunity in the susceptibility to T1D, the nucleotide-binding oligomerization domain-containing protein 2 (Nod2) has been identified as a key factor [99]. Nod2, mainly expressed in neutrophils and monocytes/macrophages, recognizes bacterial molecules which possess the muramyl dipeptide (MDP) moiety and stimulates an immune reaction, inducing CD4^+^ Th1 and CD4^+^ Th17 cells in pancreatic tissue, contributing to autoantibody production and tissue damage [100, 101].

Recently, Li et al. have generated Nod2^−/−^ nonobese diabetic (NOD) mice with a different gut microbiota composition compared to Nod2^+/+^ NOD mice. Nod2^−/−^ NOD mice appear to be significantly protected from diabetes and present a significant reduction in the proinflammatory cytokine-secreting immune cells and an increase in Tregs [99]. Interestingly, when Nod2^−/−^ NOD mice were housed with Nod2^+/+^ NOD mice, they lost the protection from diabetes, and this evidence confirmed that T1D susceptibility in Nod2^−/−^ NOD mice is dependent on the alteration of gut microbiota, which modulated the frequency and function of IgA-secreting beta-cells and IL-10 promoting T-regulatory cells. Thus, this study has confirmed the close relationship between gut microbiota and T1D susceptibility and the strong interaction between gut microbiota and the immune system.

Several studies have specifically investigated the role of adaptive immune cells in the pathogenesis of T1D. There is evidence that pancreatic islets infiltrating lymphocytes induce beta-cell damage via CD8^+^ cytotoxic T-cells. This abnormal activation is believed to be the consequence of mechanisms of molecular mimicry and of microbial infections triggering an immune response. Recent studies have focused on the possible role of TLRs. Pancreatic beta-cells express TLR4 which make them sensitive to LPS, promoting and activating transcription of NF-kB-related proinflammatory genes that mediate an immune response against microbial invasion. Thus, the upregulation of TLR4 is a further mechanism to understand the pathogenesis of T1D [71].

### 3.2. Metabolic Syndrome and Type 2 Diabetes

Metabolic syndrome is defined by a complex cluster of various elements, including visceral obesity, abnormal glucose metabolism, dyslipidaemia, and arterial hypertension. Metabolic syndrome is associated with an increased risk of type 2 diabetes (T2D) and cardiovascular diseases [102]. The disease is characterized by an increased cytokine production (mainly TNF-alpha and IL-1beta) [103], with a persistent low-grade inflammation [104]. This, in turn, generates a continuous recruitment of immune cells in metabolically active tissues, such as adipose tissue, the pancreatic gland, thyroid, liver, and muscle [105, 106]. T2D is a multifactorial disease, and several factors are involved in its pathogenesis, including diet, obesity, and gut dysbiosis [107].

Gut microbiota has conclusively been linked to the pathogenesis of both metabolic syndrome and T2D. Recently, Guo et al. developed a mouse model with high-fat feeding and demonstrated that the diet was able to alter gut microbial communities, the Paneth cell-related antimicrobial peptide production, and even to increase circulating proinflammatory cytokines, such as TNF-alpha, IL-6, and IL-1beta [108]. Thus, it is the intestinal dysbiosis related to diet, rather than adipose tissue per se, that has a pivotal role in developing intestinal inflammation.

Hence, gut microbiota by affecting the production and storage of energy could influence body weight and obesity [8], tissue proinflammatory activity, peripheral insulin resistance, pancreatic intestinal hormone production, and finally bile acid metabolism [109]. Consequently, in metabolic syndrome, the increase in the Firmicutes/Bacteroidetes *ratio* corresponds to body weight and promotes the hydrolysis of nondigestible polysaccharides in the gut, which in turn favors an increase in calories extracted from food [110, 111]. Several metagenomic studies performed on metabolic syndrome and T2D patient stools compared to healthy subjects revealed an increase in the order Lactobacillales with a decrease in *Roseburia intestinalis*, *Faecalibacterium prausnitzii*, *Bacteroides*, *Prevotella genera*, *Bifidobacterium animalis*, and *Methanobrevibacter smithii*. On the other hand, *Staphylococcus aureus*, *Escherichia coli*, and *Lactobacillus reuteri* have been found to be elevated and to predict the development of obesity [107].

Certain types of bacteria, such as *Tannerella* spp., are associated with oral infections and periodontal disease. These are typically characterized by an increase of several proinflammatory cytokines like TNF-alpha, IL-1beta, and IL-6 [112]. Gram-negative bacteria-induced LPS is able to trigger an immune response via LPS-binding protein (LBP), which in turn binds the macrophage receptor CD14. The complex formed by LPS-LBP and CD14 may activate NF-kB and AP-1 proinflammatory genes via TLR4 [113]. LPS may also activate the macrophage and dendritic cell NOD-like receptors (NLRs) that induce NF-kB in association with TLR4 [114]. In this way, a mouse model demonstrated that the lack of TLR4 protects against insulin resistance [115].

Finally, recent evidences demonstrated that intestinal dysbiosis may also mediate alterations in the Th17 cells/Tregs balance. So, the breakdown in the physiological equilibrium between pro- and anti-inflammatory T-cell subpopulations may be responsible for the development and progression of several inflammatory diseases, both in the gastrointestinal tract and in the systemic ones, including obesity-associated metabolic syndrome and T2D [104]. Thus, intestinal dysbiosis is intimately linked to significant alterations in Th17/Tregs balance contributing to obesity, metabolic syndrome, and T2D. Understanding the complex mechanisms responsible for this alteration will allow to develop novel translational therapeutic strategies to potentially treat these widespread diseases.

## 4. Pancreatic Cancer

Pancreatic cancer is extremely aggressive, with a very poor prognosis. Only 25% of pancreatic cancer can be surgically removed at the time of diagnosis. About 95% of them are adenocarcinomas that originate from gland, ductal, or acinar cells of the exocrine pancreas [116].

A link among dysbiosis, chronic inflammation, and pancreatic cancer has been well established [117–120]. Importantly, dysbiosis is considered not to have a direct mutagenic action disrupting cell cycle control, activating oncogenic signaling pathways, and producing tumor-promoting metabolites [121–124]. However, intestinal dysbiosis can activate the immune system through several pathways involving tumor-infiltrating lymphocytes (TILs) and their related cytokines, innate immune cells, TLRs, and others. In this way, TILs produce proinflammatory mediators inducing STAT3 and NF-kB pathways that act as tumorigenic factors increasing cellular proliferation and suppressing apoptosis [125–127].

Several germ-free mouse models have allowed to understand the significant impact of gut microbiome in carcinogenesis. In fact, germ-free animals have a significant reduction in cancer development, probably due to decreased gut dysbiosis and related chronic inflammation [1, 128]. In the same way, a reduction in cancer development has been observed in mice after antibiotic treatment that may be responsible for the reduction of the pathogen load in the gut mucosa [117]. Other experimental evidence has highlighted the close relationship among diet, xenobiotics, gut microbiota, and cancer [129]. In one study, mice genetically predisposed to colorectal cancer displayed increased tumor progression in a context characterized by a specific microbiota composition. This tumor-predisposing phenotype could be transferred to healthy mice after microbiota transplant using fecal samples. Interestingly, in these mice, antibiotics were able to limit tumor development, probably blocking the tumor-inducing gut microbiota [129]. However, antibiotics could also have a detrimental role. In a recent case-control study conducted on a very large cancer population, Boursi et al. proved that repeated antibiotic exposure is able to promote cancer formation, probably due to a change in microbiota [130]. This study revealed that especially the use of penicillin was associated with an elevated risk of developing colorectal, esophageal, gastric, and pancreatic cancers [130].

In chronic pancreatitis people who harbor a KRAS mutation, there is an increased risk of cancer [131, 132]. In these individuals, gut dysbiosis is able to accelerate pancreatic carcinogenesis due to the mutated KRAS hyperstimulation by the LPS-driven inflammation and by the TLR-mediated NF-kB proinflammatory gene transcription [133, 134]. The role of Gram-negative LPS-TLR4 interaction in inducing chronic inflammation and cancer has been well recognized [135]. In a recent study, Ochi et al. specifically demonstrated their impact in the pathogenesis of pancreatic cancer [136]. In a mouse model, the administration of LPS was able to significantly accelerate carcinogenic progression. On the other hand, the inhibition of TLR4 limited cancer progression, while the inhibition of the TLR adapter protein myeloid differentiation primary response gene 88 (MyD88) unpredictably worsened pancreatic inflammation and cancer development. The procancerogenetic and inflammatory actions of MyD88 inhibition are mediated by dendritic cells (DCs), which were able to induce pancreatic antigen-restricted Th2 cells and promote the transition from pancreatitis to pancreatic cancer [136].

Pathogens are able to act as carcinogenetic agents after infecting the pancreatic gland through intestinal translocation. Among these, a special role is played by *Helicobacter pylori* [72]. In fact, it has been well established that it may promote the carcinogenesis of the stomach, liver, and pancreas, by inducing the activation of the nuclear factor NF-kB and its proinflammatory cytokines, such as IL-1beta [137]. *Fusobacterium* species have also been linked to the development of pancreatic cancer, and they are associated with worse prognosis [138].

Recently, Ren et al. studied the microbiota profile of 85 pancreatic cancer patients compared to 57 healthy people [139]. This study revealed that gut microbial diversity is significantly reduced in pancreatic cancer and this tumor is characterized by a unique microbial profile. In particular, the microbial alterations in pancreatic cancer regarded an increase in several pathogens, such as *Veillonella*, *Klebsiella*, and *Selenomonas*, and LPS-producing bacteria including *Prevotella*, *Hallella*, and *Enterobacter*, and a related decrease in several commensals, such as *Bifidobacterium*, and some butyrate-producing bacteria, such as *Coprococcus*, *Clostridium* IV, *Blautia*, *Flavonifractor*, and *Anaerostipes* [139]. The evidence of the increase in the LPS-producing bacteria confirms the role of dysbiosis in mediating chronic inflammation and oxidative damage activating the NF-kB pathway and its related proinflammatory cytokine production. In this way, long-standing chronic inflammation and oxidative damage participate in the development of cancer.

Likewise, it has been shown that pancreatic cancer is associated with an alteration of the physiological oral microbiota composition [140]. Oral microbiota is composed of more than 700 bacteria species which contribute to health and physiology of the mouth, teeth, and oral cavity [117]. Alterations in the taxa dominance and diversity among oral microbial communities, particularly regarding those related to the periodontal disease, may be associated with an increased pancreatic cancer risk [140]. Farrell et al. performed a study analyzing salivary microbiota of several pancreatic cancer and chronic pancreatitis patients compared to healthy subjects [141]. These authors demonstrated that pancreatic cancer is related to a specific alteration in salivary microbiota composition. In particular, it was shown that *Neisseria elongata*, *Corynebacterium* spp., and *Streptococcus mitis* decreased, while *Granulicatella adiacens* and *Porphyromonas gingivalis* increased [140, 141]. Recently, Torres et al. conducted a cross-sectional study showing an increase in *Leptotrichia* spp. and a reduction in *Porphyromonas* spp. in pancreatic cancer patient saliva; thus, a higher *Leptotrichia : Porphyromonas* (L : P) ratio may become an important pancreatic cancer diagnostic biomarker [142]. Otherwise, Michaud et al. demonstrated that high antibody titer against gut commensal bacteria was associated with a reduction of 45% in the risk of pancreatic cancer compared to those with a lower antibody titer [143]. In the same way, these authors revealed that the highest concentration of serum antibodies to the pathogenetic bacteria *Porphyromonas gingivalis* (associated with periodontal disease) was linked to a 2-fold increased risk of pancreatic cancer [143].

Altogether, these evidences highlight the potential to develop future novel diagnostic tools to detect early pancreatic cancer, utilizing samples easy to collect, such as blood, saliva, and stools. However, at the present time, it is not possible to discriminate whether these gut microbial alterations exert a causal role in the developing of pancreatic cancer or, instead, are a result of cancer formation.

Importantly, it should be noted that chronic inflammation-related pancreatic cancer development may occur even without the presence of bacteria. This type of sterile inflammation may be triggered by distant intestinal dysbiosis or translocation of bacteria components, such as LPS, and it is guided by the activation of the immune system through TLRs. In this way, TLR2, TLR4, and TLR9 have been recently shown to be associated with pancreatic cancer development [144, 145].

Finally, recent evidences have shown that gut microbiota and antibiotics may alter tumor response to chemotherapy by modulating tumor microenvironment [146, 147]. Hence, gut microbiota may modify the efficacy of traditional cancer chemotherapies, the novel immune-target drugs, such as anti-CTLA4 and anti-CD274 therapies, but also the tumor recurrence after pancreatic surgery [121].

In conclusion, pancreatic cancer is considered a very insidious and aggressive disease characterized by late diagnosis and no effective screening methods. In this way, in the one hand, it may be too early to hope in the routine use of gut microbiome modulation for therapeutic purposes, and on the other hand, gut microbiome profiling may have important diagnostic tools in the prediction of pancreatic cancer development, thus improving the survival rates associated with this disease.

## 5. Conclusions

Gut microbiota is central to the development and modulation of the intestinal homeostasis and mucosal immune system integrity and exerts an important role in the protection against pathogenic microbes by maintaining gut integrity and regulating intestinal barrier permeability.

The pancreas does not possess its own microbiota, and the available evidence demonstrates that alteration of gut microbiota determining dysbiosis and bacterial translocation (Table 1) is correlated with the duration and prognosis of several pancreatic disorders, including pancreatitis, diabetes, and cancer. However, whether gut dysbiosis is the cause or an effect of such pathological conditions remains unclear.

In principle, the pharmacological modulation of gut microbiota may be beneficial in the treatment of pancreatic conditions and related complications. However, the use of prebiotics, probiotics, antibiotics, and anti-inflammatory drugs or the fecal microbiota transplantation either as a preventative or as a therapeutic strategy remains controversial. These procedures have not yet been a subject to the rigorous efficacy and safety testing necessary to recommend their routine use.

In the foreseeable future, the analysis of specific alterations in the microbiome profile may permit to develop novel tools for the early detection of several pancreatic disorders, utilizing samples easy to collect, such as blood, saliva, and stools.

In conclusion, the ways in which gut microbiota is modulated and interacts with the immune system need to be further elucidated to enter a new era of treatment modalities.

## Figures and Tables

**Table 1:** Gut microbiota alterations in pancreatic pathologies.

*Bacterium* (phylum)	Acute pancreatitis [28]	Chronic pancreatitis [27]	Autoimmune pancreatitis (AIP)	Type 1 diabetes (T1D)	Metabolic syndrome and type 2 diabetes (T2D)	Pancreatic cancer
Increase	*Enterococcus* spp. (Firmicutes) Enterobacteriaceae	Firmicutes	*Helicobacter pylori* (**Molecular mimicry** mechanism) [72–75] *Escherichia coli* (**Trigger** mechanism) [75]	*Bacteroides dorei* and *vulgates* (Bacteroidetes) [38, 82] *Klebsiella* spp. (Enterobacteriaceae) [89] *Coxsackievirus* A and B, *Echovirus*, *Enterovirus* [83]	Lactobacillales, *Staphylococcus aureus* (Firmicutes) [107] *Escherichia coli* (Proteobacteria) [107] *Tannerella* spp. (Bacteroidetes) [112]	*Helicobacter pylori* (Proteobacteria) [72] *Fusobacterium* [138] *Leptotrichia* [142] (Fusobacteria) *Veillonella* spp. *Selenomonas* spp. (Firmicutes) [139] *Klebsiella* spp. *Enterobacter* spp. (Enterobacteriaceae) [139] *Prevotella* spp. *Hallella* spp. (Bacteroidetes) [139] **Salivary microbiota:** [140, 141] *Granulicatella adiacens* (Firmicutes) *Porphyromonas gingivalis* (Bacteroidetes)

Decrease	Bacteroidetes *Bifidobacterium* spp. (Actinobacteria) *Lactobacillus* spp. *Clostridium cluster XI* (Firmicutes)	Bacteroidetes *Faecalibacterium prausnitzii* (Firmicutes) *Ruminococcus bromii* (Firmicutes)	—	*Lactobacillus* spp. *Clostridium leptum* (Firmicutes) [38, 82] *Bifidobacterium* spp. (Actinobacteria) [91] *Prevotella* spp. (Bacteroidetes) [89] *Akkermansia muciniphila* (Verrucomicrobia) [89]	*Bacteroides* *Prevotella* (Bacteroidetes) [107] *Roseburia intestinalis* *Faecalibacterium prausnitzii* (Firmicutes) [107] *Bifidobacterium animalis* (Actinobacteria) [107] *Methanobrevibacter smithii* (Methanobacteria) [107]	*Bifidobacterium* spp. (Actinobacteria) [139] *Coprococcus* spp. *Clostridium cluster IV* *Blautia* spp. *Flavinofractor* spp. (Firmicutes) [139] **Salivary microbiota:** [140, 141] *Neisseria elongata* (Proteobacteria) *Streptococcus mitis* (Firmicutes) *Corynebacterium* spp. (Actinobacteria)
